# Interaction of Diphenhydramine Hydrochloride with Cationic and Anionic Surfactants: Mixed Micellization and Binding Studies

**DOI:** 10.3390/polym13081214

**Published:** 2021-04-09

**Authors:** Naved Azum, Malik Abdul Rub, Sulaiman Yahya Alfaifi, Abdullah M. Asiri

**Affiliations:** 1 Chemistry Department, Faculty of Science, King Abdulaziz University, Jeddah 21589, Saudi Arabia; salfaifi@kau.edu.sa (S.Y.A.); aasiri2@kau.edu.sa (A.M.A.); 2 Center of Excellence for Advanced Materials Research, King Abdulaziz University, Jeddah 21589, Saudi Arabia; malikrub@gmail.com

**Keywords:** diphenhydramine hydrochloride, sodium dodecyl sulfate, cetylpyridinium chloride, mixed micellization, binding studies

## Abstract

The focus of the present work is to evaluate the interactions of an anti-allergic drug (diphenhydramine hydrochloride, DPH) with anionic (sodium dodecyl sulfate, SDS) and cationic (cetylpyridinium chloride, CPC) surfactants in the aqueous medium. The mixed micellization behavior and surface properties of drug-surfactant mixtures have been examined by surface tension measurements. Various theoretical approaches were applied to explore the synergistic or non-ideal behavior of the current mixed systems. Furthermore, the binding studies of drug with surfactants have been elaborated by UV–visible spectroscopy. Benesi–Hildebrand (B-H) theory was used to compute stoichiometric ratio, binding constant, and free energy change for the drug-surfactant mixtures. The outputs are deliberated taking into consideration the use of surfactants as capable drug delivery agents for DPH and hence advance bioavailability.

## 1. Introduction

A pharmaceutical drug or medicine is a substance that causes physiological changes in the human body and is used to treat or cure a disease. To study physicochemical properties of the drug is of most importance to understand the drug action at the molecular level which is highly dependent upon the solution behavior of the drug. Many drugs used in pharmaceutical formulations or drug delivery are surface-active drugs [1,2,3,4,5,6,7,8,9,10,11,12,13,14,15,16,17,18,19,20,21]. The surface-active agents are organic molecules having both hydrophilic and hydrophobic parts in the same body. The solvent being polar is attracted by the hydrophilic part while the hydrophobic part composed of long carbon chains favors the hydrophobic interaction. The presence of two opposite nature parts in the same body, allows these molecules to adsorb at the surface or interface and reduce the surface tension of the examine solution. The applications of these molecules are vast and in almost every field including household products, pharmaceuticals, biomedical, food industries, and cosmetics [22]. In pharmaceuticals the use of surface-active agents increases due to its self-assembling nature. These surface-active agents can act as a solubilizer, stabilizers, and dispersion agent in pharmaceuticals. They are also used as an antibacterial agent. They are also used as an excipient to improve the drug formulation of antibiotics. Drug because of amphiphilic in nature or surfactant-like structure they form small organized aggregates, micelle at a specific concentration known as critical micelle concentration (CMC). Micelle is a key point to provide various functional properties of the drug in the presence of different pharmaceutical excipients.

The bioavailability and therapeutic efficacy of the drug depend on solubility [23,24,25]. According to Khadka et al. [26] almost 70% of new chemical entities discovered for pharmaceutical applications have poor water solubility. The less soluble drug gives unsatisfactory and variable consumption while excess drug doses lead to several side effects. Hence, various strategies have been applied to improve the solubility of the drug, including the use of surfactants. The surfactants are widely used in drug delivery and the mixed system composed of drug and surfactants are better competent as compared with their single components counterparts. The main action of surfactant is the transport of the drug to the specific sites; thus, they lessen the drug degradation and enhance bioavailability. The surfactant molecule being the imitate to bio membrane help in studying the interaction of the different drug with a membrane.

**Figure 1:**
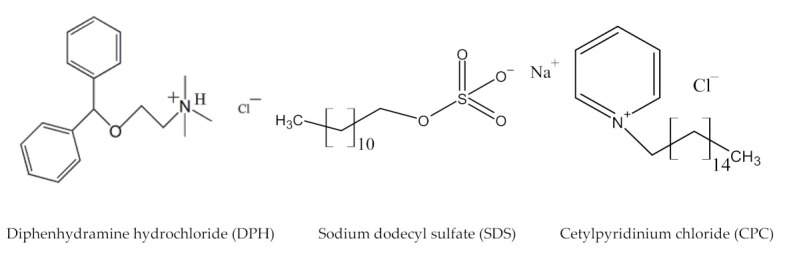
Structure of amphiphiles.

Diphenhydramine (DPH) is an ethanolamine, an antihistamine of H_1_ receptor, a first-generation drug, and has anticholinergic properties [27,28,29]. The reported value of pKa for DPH is 8.87, so the DPH ionized in an aqueous solution and acquired a positive charge. We checked the pH of the pure and mixed solution and found a constant value ≈ 7.0. DPH works by blocking the effects of histamine a natural substance produces in the body during an allergic reaction. Histamines play a major role in mediating nasal allergy symptoms, such as mucus formation, swelling, and itching [30]. Some other conditions like hay fever, nausea, vomiting, and dizziness are also controlled by using this drug. It is found in many nonprescription products as the sole active ingredient or combined with other ingredients for the treatment of colds, allergies, and insomnia. It is available in oral tablets, chewable tablets, liquid-filled capsules, and liquid solution forms. It is also used on the skin in the form of topical anti-itch cream, gels, and sprays. Being first-generation DPH has more efficacy in the treatment of allergies than some second-generation antihistamine drug Desloratadine. The surface activity of this compound is due to the presence of the diphenylmethane group (Figure 1).

The main aim of this work is to investigate the interaction of anti-allergic drug (DPH) with anionic (SDS) and cationic (CPC) surfactants. The micellar compositions, self-aggregation, mutual interaction, interfacial adsorption, and thermodynamic of the mixed system were studied in detail. Different physical theories of mixed micelle like Rosen, Rubingh, Clint, and Motomura have been used to understand the interactions between drug and surfactant components. Furthermore, the binding studies of drug with surfactants have been elaborated by UV–visible and steady-state fluorescence.

## 2. Experimental Section

### 2.1. Materials

Sodium dodecyl sulfate (SDS), cetyl pyridinium chloride (CPC) were the product from Sigma-Aldrich (St. Louis, MO, USA) having purity >98%, while diphenhydramine hydrochloride (DPH), was purchased from Molecule-on (New Lynn, Auckland, New Zealand). The structures of all amphiphiles are shown in Figure 1. All chemicals were used as provided by the supplier without further purification. Ultrapure deionized double distilled water has been used to make the solution of amphiphiles and their mixtures.

### 2.2. Methods

#### 2.2.1. Surface Tension Measurement

**Figure 2:**
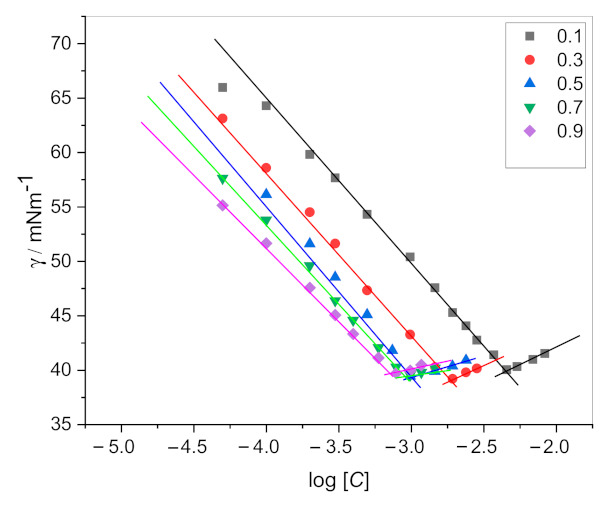
Surface tension (γ) vs. log [*C*] plots for CPC + DPH mixed systems.

The surface tension values (γ) of drug, surfactants, and their mixtures were recorded by using an attension tensiometer (Sigma 701, Attension, Germany) over the temperature of 298.15 K. We have followed the ring method (Du Nouy principle) to measure the surface tension. According to Du Nouy principle the force is measured before a liquid film is tear using a torsion meter and surface tension calculated from the diameter of the ring and the tear-off force. The instrument was calibrated with double distilled ultrapure water having surface tension value (γ) = 70 mN·m^−1^ at 298.15 K. Before each measurement, the ring was heated on an ethanol flame until glowing red and finally cleaned by washing in water. The γ values for pure and mixtures of amphiphiles were obtained by adding concentrated amphiphiles solution in the vessel containing water. The γ values continuously declined on the adding of amphiphiles solution up to a specific value of concentration and then it became constant. This constant value of concentration refers to the CMC value, as shown in Figure 2. Surface tension data is replicated three times for each experiment.

#### 2.2.2. Electronic Absorption Measurement

**Figure 3:**
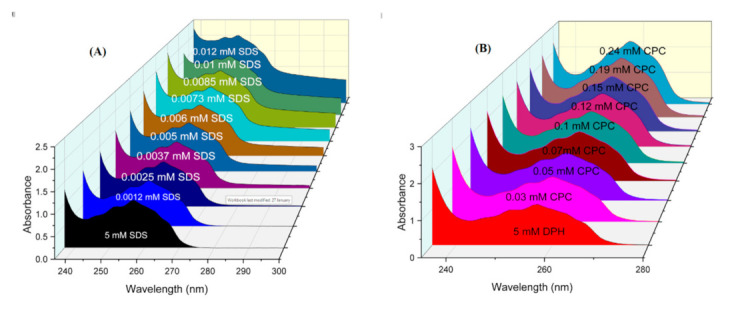
Adsorption spectra of DPH with increasing concentration of (**A**) SDS, (**B**) CPC.

UV–visible spectrophotometer (Thermo Scientific, Waltham, MA, USA, Evolution 300) was used to obtained absorption spectra of DPH in the presence of SDS and CPC using a quartz cuvette. The measurements were done to recognize the nature of the interaction between the drug and surfactants. Benesi–Hildebrand equation was used to compute the binding constant (*K*). The absorption spectra of DPH have been recorded over 200 to 400 nm (Figure 3). For absorption of drug surfactant mixture, increasing equivalents of surfactants were added by keeping the concentration of drug constant (Figure 3).

## 3. Results and Discussion

### 3.1. Critical Micelle Concentration of Single and Mixed Amphiphiles

**Figure 4:**
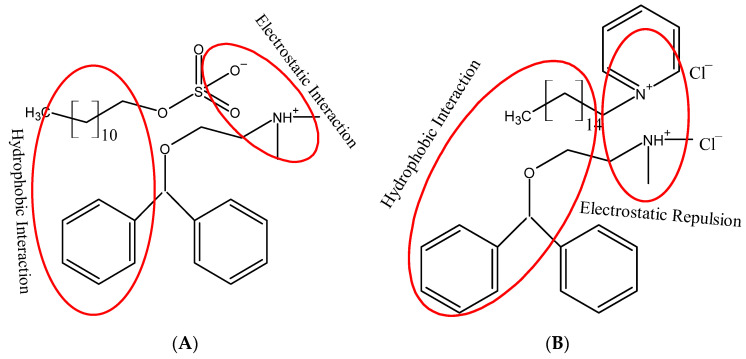
Schematic representation of the interactions (hydrophobic and electrostatic) between (**A**) DPH + SDS, (**B**) DPH + CPC.

The critical micelle concentration of single and mixed amphiphiles has been determined from surface tension vs. concentration plots (Figure 2). CMC plays a significant role in many product formulations in various industries. When the interface between the two-phase is saturated with the amphiphile monomers, the formation of micelle in the bulk is taking place naturally. When we add an amphiphile to the water or solvent, the interfacial tension (force per unit length, mN/m) decreases. When the amphiphile molecules adsorb at the interface, they replace some water molecules. The amphiphile–water interaction is weaker than the water–water molecular interaction, results in contraction force decreases (surface tension decreases). At the saturation point (CMC), the amphiphile starts to aggregate and form a globular structure known as a micelle. Exploring the CMC values of an amphiphile is very crucial as it is an important indicator where it can effectively emulsify, solubilize, and disperse. Determining the CMC via surface tension is one of the classical ways. Figure 2 is showing a graph between surface tension vs. concentration of amphiphile, the CMC is determined when the surface tension becomes almost constant. The CMC values of solo and mixture of amphiphiles determined by the surface tension are presented in Table 1. The CMC values of single amphiphiles are well-matched with earlier reported values [31,32,33]. The presence of an aromatic ring and 16 carbon in the chain of the CPC molecule make the CMC less than SDS. The DPH has a higher CMC value due to the rigid structure having two rings. So, it is tough for DPH molecule to fit in the curved area for the formation of the CMC. Therefore, the formation of micelle takes place at a higher concentration. Usually, the CMC values for mixtures are found between the CMC values of the two amphiphiles [34,35,36,37]. The CMC value of DPH is decreased in the presence of SDS and CPC. The decrease in CMC with the addition of surfactants suggests a synergistic interaction between the drug and surfactant molecules. Generally, the hydrophobic interaction between the chains and electrostatic repulsion between the head groups decide the onset of micelle formation. A micelle is formed when hydrophobic interaction in the system predominates over electrostatic repulsion. For SDS+DPH mixed system the CMC values are lower than the individual amphiphiles (except α_1_ = 0.1). The electrostatic repulsive interactions between SDS ions are reduced by the addition of drug molecules through the electrostatic attractive interactions between (SO_4_) group of SDS and NH^+^ group of dug molecules (Figure 4). Hence, as a result, monomers aggregate more easily and CMC values of DPH decreases in the presence of SDS. In the case of CPC+DPH both have a positive charge on the head group. The hydrophobic interactions may overcome the electrostatic repulsions between the cationic head group of CPC and DPH, and an increase in the hydrophobicity may cause the drug molecules to move slightly deeper into the micelle interior. The decrease of CMC of the drug in the presence of CPC (having both positive charge head groups) favored the increase in the hydrophobic interaction and decrease in the electrostatic self-repulsion.

To judge the interaction between two amphiphiles, theoretical models were used. The ideal and nonideal solution behavior of amphiphile mixtures can be described by using the pseudo-phase separation model. The ideal values of CMC (CMC*) can be assessed with the help of experimental CMC values of single amphiphiles using Clint’s relations [38,39]:

(1) $$\frac{1}{{\mathrm{CMC}}^{*}}=\;\frac{\alpha_{1}}{{\mathrm{CMC}}_{1}}+\;\frac{\alpha_{2}}{{\mathrm{CMC}}_{2}}$$

CMC* = ideal critical micelle concentration of the mixed system
CMC_1_ = critical micelle concentration of surfactant (SDS/CPC)
CMC_2_ = critical micelle concentration of the drug (DPH)
α_1_ = mole fraction of surfactant (SDS/CPC)

The ideal value of CMC (CMC*) can be predicted from the CMC values of single amphiphiles by this model. It helps to know the ideality of the mixtures. In Table 1, experimental CMC and ideal CMC are listed. It is confirmed from Table 1 that the obtained CMC values are seen to be less than CMC* values, showing a negative deviation from ideality. The deviation from ideality refers to the interactions between the two amphiphiles. If the deviation is negative (CMC < CMC*), represent attractive interaction; deviation is positive (CMC* < CMC), show repulsive interaction. Since both systems show negative deviation from ideality, hence an attractive interaction exists between the drug and surfactant molecules.

### 3.2. Synergistic Effects

Although Clint equation is helpful to know the non-ideality of the mixture. But when the two amphiphiles of different head groups are mixed, the interaction of the mixed system cannot be predicted accurately by Clint’s equation. To solve this problem Rubingh [40,41,42] gave a relation by applying regular solution approximation (RST). Rubingh theory is one of the most frequently used theories among several others to get an insight into the micellar constitution. Rubingh proposed a relation between CMC values of single and mixed amphiphiles and stoichiometric mole fraction to compute the values of micellar mole fraction of amphiphiles in the mixture (*X*_1_).

(2) $$\frac{{\left(X_{1}\right)}^{2}ln\left(\alpha_{1}CMC/X_{1}CMC_{1}\right)}{{\left(1-X_{1}\right)}^{2}ln\left[\left(1-\alpha_{1}\right)CMC/\left(1-X_{1}\right)CMC_{2}\right]}\;=1$$

The values *X*_1_ can be obtained by solving Equation (2) iteratively. According to Motomura approximation [43,44], the micellar mole fraction in the ideal state is also computed by using the following equation:

(3) $$X_{1}^{\mathrm{ideal}}=\frac{\alpha_{1}CMC_{2}}{\alpha_{1}CMC_{2}+\alpha_{2}CMC_{1}}$$

The values of both *X*_1_ and $X_{1}^{\mathrm{ideal}}$ are listed in Table 1. It is confirmed from the data that the $X_{1}^{\mathrm{ideal}}$ values are higher than *X*_1_, for both mixed systems. This suggests the contribution of surfactants is lower than the drug in the formation of mixed micelles.

Another interaction parameter (*β*) is also important to understand the nature and magnitude of the interaction between two components. By using the calculated values *X*_1_, the micellar interaction parameter (*β*) can be obtained by the given relation,

(4) $$\beta =\frac{\ln\left(\alpha_{1}CMC/X_{1}CMC_{1}\right)}{{\left(1-X_{1}\right)}^{2}}$$

The values of *β* may be positive, negative, or even zero. The *β* with negative values imply synergism arising from attractive interactions, whereas the *β* with positive values indicated antagonism because of the repulsive interaction between the amphiphiles molecules in the micellar phase. It is also a measure of any deviation from ideal behavior. The values of *β* are listed in Table 1 and are found to be negative for both mixed systems. The negative values of *β* indicate synergistic interaction. The SDS+DPH shows greater synergism than CPC+DPH. This can be clarified based on fact that the DPH binds tightly with SDS because of preferred electrostatic interactions amid the cationic head group of DPH and anionic head group pf SDS, which form ion pair. For synergism between two amphiphiles two circumstances must be trailed:

(1) *β* should be negative
(2) $\left|\beta \right|>\left|\ln(CMC_{1}/CMC_{2}\right|$

It is confirmed from Table 1, both conditions are followed by all binary mixtures studied. The evaluated values of *β* were used in Rubingh’s equation to calculate the activity coefficients

(5) $$f_{1}=exp\left[\beta {\left(1-X_{1}\right)}^{2}\right]$$

(6) $$f_{2}=exp\left[\beta {\left(X_{1}\right)}^{2}\right]$$

The calculated values are less than one which signifies the synergistic or non-ideal behavior between drug and surfactant mixtures at all mole fractions.

### 3.3. Surface Parameters of Drug-Surfactants Mixtures

The unbalance attractive force between the surface molecules (force per unit of length) is known as surface tension or surface energy. When amphiphiles are adsorbed at the surface, some water molecules are replaced by the amphiphiles. The amphiphiles–water molecules interaction is weaker than water–water molecular interactions, resulting in contraction force decrease (surface tension decreases). The adsorption per unit area of amphiphiles can be calculated by the Gibbs adsorption Equation (7):

(7) $$-d\gamma =\sum \Gamma_{i}d\mu_{i}$$

where

γ = surface tension
Γ*_i_* = surface excess
*μ_i_* = chemical potential of the *i*th components in the solution

For a multi-component system having *n* species and total concentration *C*, the equation may be written as [45,46,47]

(8) $$\Gamma_{max}=-\frac{1}{2.303nRT}\left(\frac{d\gamma }{dlogC}\right)$$

where *n* represents the number of species present in the solution, and its value can be calculated by the relation [20]

$$n\;=\;X_{1}^{S}n_{1}\;+\;X_{2}^{S}n_{2}$$

The values of $\Gamma_{max}$ can be used to calculate the minimum area per surfactant molecules, at the air/water interface by using the Equation (9) [31,48]

(9) $$A_{min}=\frac{10^{18}}{N_{A}\Gamma_{max}}$$

where *N*_A_ stands for Avogadro’s number. The packing of molecules at the surface, whether the molecules are closely or loosely packed at the surface, are recognized by the *A_min_* values. The values of these parameters are listed in Table 2. The values of $\Gamma_{max}$ decreases (*A_min_* increases) in the presence of CPC while the values increase (*A*_min_ decreases) in the presence of SDS. The lower values of $\Gamma_{max}$ for DPH+CPC mixed system than pure DPH is due to the larger hydrocarbon chain of CPC that resists the adsorption of the mixture on the surface. This is further confirmed by the values of *A_min_*. The larger values $\Gamma_{max}$ for SDS+DPH mixed system than $\Gamma_{max}\;$ value of DPH indicating that the SDS+DPH mixed system possesses more adsorbing tendency at the surface and favors the micellization in the bulk solution. The lower values of *A_min_* for the SDS+DPH mixed system are accountable for close packing. The higher values of *A_min_* for CPC+SDS mixed system than the value for pure DPH indicating the formation of loose packing and micelle with a relatively high charge density of CPC and DPH mixed system. The minimum area per molecule at an ideal state (*A_ideal_*) can be computed from Equation (10)

(10) $$A_{ideal}=X_{1}^{s}A_{1}+\left(1-X_{1}^{s}\right)A_{2}$$

where $X_{1}^{s}$ is the mole fraction of component 1 at the surface. It is confirmed from Table 2, that the values of $A_{ideal}$ for both mixed systems are lower than the *A_min_* experimental values. The *A_min_* values are also utilized to compute the packing parameter (*p*), the geometry of micelle by the following Equation (11) [49]:

(11) $$p=\frac{V_{0}}{l_{c}A_{min}}$$

where *V*_0_ = volume of exclusion per monomer in the aggregate, *l*_c_ = is the maximum chain length computed by the Tanford formulae [50]

(12) $$V_{0}=\left(0.0274+0.0269\;\left(C_{n}-1\right)\right)\;nm^{3}$$

(13) $$l_{c}=\left(0.154+0.1265(C_{n}-1)\right)nm$$

The values of *p* are listed in Table 3. Spherical, rod and disk-like are some existing morphologies of an amphiphile. Due to these different morphologies, the different structures like cubic, lamellar, and cage are possible depending on the *p*-value. The *P* values for spherical, cylindrical, and lamellar particles are ~0.33, 0.33 to 0.5, and 0.5 to 1, respectively. From Table 3 the *p* values of pure and mixtures are below 0.33, confirm spherical geometry.

To know the solution behavior of an amphiphile, the efficiency of surface adsorption is the most important criterion. It is defined as the required molar concentration of an amphiphile to produce maximum adsorption. Generally, when the surface tension value of an amphiphile is reduced to 20 mN·m^−1^, maximum adsorption is acquired. The molar concentration of an amphiphile at this point is known as *C*_20_ and its negative logarithm value is symbolized as *pC*_20_. The larger *pC*_20_ values mean better efficiency of amphiphiles is to be adsorbed. For both mixed systems the *pC*_20_ is higher than pure DPH (Table 2), indicating that the mixed systems have a higher adsorption tendency at the surface relative to the solubilizing tendency in an aqueous medium.

The nature and strength of the interaction of the drug with surfactant at the surface can be analyzed by using Rosen’s theory [51]. The micelle mole fraction at mixed adsorbed film and interfacial micellar interaction parameter between two amphiphiles can be evaluated by the following equations:

(14) $$\frac{{\left(X_{1}^{S}\right)}^{2}\ln\left(\alpha_{1}C^{S}/X_{1}^{S}C_{1}^{S}\right)}{{\left(1-X_{1}^{S}\right)}^{2}\ln\left[\left(1-\alpha_{1}\right)C^{S}/\left(1-X_{1}^{S}\right)C_{2}^{S}\right]}\;=1$$

(15) $$\beta^{S}=\frac{\ln\left(\alpha_{1}C^{S}/X_{1}^{S}C_{1}^{S}\right)}{{\left(1-X_{1}^{S}\right)}^{2}}$$

where *C*_1_, *C*_2,_ and *C* are molar concentration values of SDS/CPC, DPH, and their mixed monolayer, respectively, α_1_ is the mole fraction of SDS/CPC. Equation (14) was solved iteratively for $X_{1}^{S}$, which is then substituted in Equation (15) to calculate $\beta^{S}$ values. As seen from Table 3. The values of $X_{1}^{S}$ increases with increasing the surfactant concentration for both mixed systems, which suggests that more surfactant molecules are adsorbed to make a mixed monolayer. The $\beta^{S}$ values reflect the extent of interaction between drug and surfactant at the surface. The $\beta^{S}$ values given in Table 3, are negative with the respective average values (–14.66 and –5.32) being for SDS/DPH and CPC/DPH. The negative values suggesting attractive interactions in mixed monolayer formation. Like bulk at the surface for synergism following two conditions should exist:

(1) $\beta^{S}$ should be negative
(2) $\left|\beta^{S}\right|>\left|\ln\left(\frac{C_{1}}{C_{2}}\right)\right|$

It is clear from Table 3 that SDS+DPH mixed system follow the conditions. It is also confirmed from data that $\beta^{S}$ > *β*, which means attractive interactions at the surface are stronger than in bulk.

### 3.4. Thermodynamic Parameters of Micellization

In the micelle formation, the ordered water structure break by the hydrophobic part of the amphiphiles, and entropy of the system increases while there is a decrease in the free energy. The attainment of a minimum free energy state is the main reason for the micelle formation. The decrease in the free energy of any process describes spontaneity. Thus, the formation of the micelle is a spontaneous process. With the help of the phase separation model of micellization, the standard Gibbs free energy of micellization ($∆G_{m}^{0}$) is calculated from Equation (16) [47].

(16) $$∆G_{m}^{0}=RTlnX_{CMC}$$

All values of $∆G_{m}^{0}$ obtained for both mixed systems are negative (Table 4), suggesting the feasibility of the micellization. The $∆G_{m}^{0}$ values of DPH are less than the pure amphiphiles (SDS and CPC) suggesting micellization of the drug is less spontaneous than the other two amphiphiles. The spontaneity of the DPH/CPC mixed system is slightly more than DPH/SDS. The result is possible because SDS is less hydrophobic than CPC, longer chain molecules favor micellization to a large degree. The synergistic interaction between two amphiphiles-like interaction parameters also quantified by the term free energy of micellization. The values (listed in Table 4) are increases with increasing the SDS/CPC concentration, suggesting synergistic interactions between DPH+SDS/CPC.

Rosen and Arason gave a simple relation between $∆G_{m}^{0}\;$ and $∆G_{ad}^{0}$ (standard gives the energy of adsorption) [51,52]:

(17) $$∆G_{ad}^{0}=∆G_{m}^{0}-\frac{\pi_{CMC}}{\Gamma_{max}}$$

The π_cmc_ is the surface pressure at CMC. The above equation is significant because it describes the transfer of an amphiphile molecule from the surface to bulk at zero pressure. The values of $∆G_{ad}^{0}$ for pure and mixed systems are also negative indicate spontaneity of adsorption. The more negative values of $∆G_{ad}^{0}$ than the $∆G_{m}^{0}$, confirm the adsorption process is more favorable than micellization.

The synergism at adsorbed monolayer evaluated by a thermodynamic parameter (*G*_min_) [53]

*G*_min_ = *A*_min_γ_CMC_N_A_

The values of *G*_min_ are listed in Table 4. The low values of *G*_min_ for pure and mixed systems indicate that the thermodynamically stable surfaces are formed, and hence drug–surfactant interactions are favorable.

The activity coefficients could be used to determine another thermodynamic parameter, the excess free energy of micellization based on Rubingh’s theory [54]:

(18) $$\Delta G_{exc}=RT\left(X_{1}lnf_{1}+X_{2}lnf_{2}\right)$$

All the calculated values of $\Delta G_{exc}$ are negative, indicating thermodynamically stable micellization takes place.

### 3.5. Spectroscopic Method (UV–Visible Spectroscopy)

**Figure 5:**
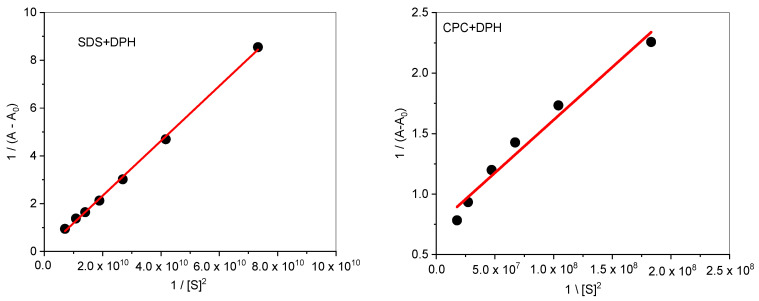
The plot of 1/(A–A_0_) vs. 1/[S]^2^ for the interaction of DPH with SDS and CPC in an aqueous solution.

When a drug is solubilized in a surfactant solution, the location, distribution, and orientation of the drug in the micelle are of great importance to know the physicochemical behavior of the drug. The interaction of drug (DPH) with anionic and cationic surfactants has been analyzed by UV–visible spectroscopy. The absorbance spectra of DPH have been analyzed in the presence of increasing equivalents of surfactants (Figure 3). It is important to note here that surfactants have no peak in the UV–visible range. There is only one characteristic peak of DPH at 257 nm, a characteristic peak of the tricyclic region of the drug. On the addition of surfactants (SDS/CPC), the absorption intensity of DPH increases. The hyperchromic effect in the drug surfactants mixture indicates the formation of a new complex. As it is clear from the figure there is no significant shifting in the spectrum so could not explain much about the behavior of the complex. The Benesi–Hildebrand Equation (19) was used to determine the quantitative estimation of the binding of surfactants with the drug [55].

(19) $$\frac{1}{A-A_{0}}=\frac{1}{K\left(A_{max}-A_{0}\right){\left[S\right]}^{n}}+\frac{1}{A_{max}-A_{0}}$$

where (*S*) is the concentration of surfactants (SDS/CPC), *A*, *A*_0_ and *A*_max_ are the absorbance values of DPH in the presence of surfactant, in the absence of surfactant, and the absorbance due to the formation of the drug–surfactant complex. The plot of $\frac{1}{A-A_{0}}$ vs. 1/[S]^2^ gives a straight line (Figure 5), which further indicates the formation of the 1:2 complex. The Bensei–Hildebrand equation was used to calculate the binding constant from intercept to slope ratio. The obtained binding constant values (K) are 3.349 × 10^8^ and 8.469 × 10^7^ M^−2^ for DPH+SDS and DPH+CPC, respectively. It is clear from the binding constant values that the DPH is showing more binding affinity toward SDS than CPC due to the less steric hindrance caused by SDS single-chain hydrophobic group. The values of K is used to calculate the values of free energy change by the Equation (20) [56].

(20) $$∆G_{K}=-RTlnK$$

The values of free energy change are −48.63 and −45.249 kJ·mol^−1^ for DPH+SDS and DPH+CPC, respectively. The values of $∆G_{K}$ were found to be negative to confirm the spontaneity of the process of complexation.

## 4. Conclusions

The mixed micellization of the anti-allergic drug, diphenhydramine hydrochloride, with anionic (SDS) and cationic (CPC) surfactants, has been studied by surface tension and UV–visible spectroscopy. This study is of great importance from a biological and pharmaceutical point of view. The outputs are deliberated taking into consideration the use of surfactants as capable drug delivery agents for DPH and hence advance bioavailability. The mixed micelle, interfacial and thermodynamic parameters were evaluated by surface tension measurement while binding constant and free energy change were evaluated by UV–visible spectroscopy. The following conclusions are drawn from our results:

(1) Experimental CMC values are seen to be less than CMC* values, showing a negative deviation from ideality. The deviation from ideality refers to the interactions between the two amphiphiles.
(2) The values micelle mole fraction of surfactants (*X*_1_) are lower than ideal values ($X_{1}^{\mathrm{ideal}}$) at all mole fractions, confirm a higher contribution of the drug in the mixed micelle.
(3) The calculated values of the interaction parameter (*β*) are negative suggesting synergistic interaction between the drug and surfactants. The SDS+DPH mixed system shows greater synergism than CPC+DPH. This can be clarified based on fact that the DPH binds tightly with SDS because of preferred electrostatic interactions amid the cationic head group of DPH and anionic head group pf SDS, which form ion pair.
(4) The lower values of surface excess ($\Gamma_{max}$) for DPH+CPC mixed system than pure DPH is due to the larger hydrocarbon chain of CPC that resists the adsorption of the mixture on the surface.
(5) The packing parameter (*p*) values of pure and mixtures are below 0.33, confirm spherical geometry.
(6) The changed standard Gibbs free energy ($∆G_{m}^{0}$) obtained for both mixed systems are negative, suggesting the feasibility of the micellization.
(7) The DPH is showing more binding affinity toward SDS (3.349 × 10^8^) than CPC (8.469 × 10^7^) due to the less steric hindrance caused by SDS single-chain hydrophobic group.

## Figures and Tables

**Table 1:** Micellar parameters, experimental critical micelle concentration (CMC), ideal critical micelle concentration (CMC*), micellar mole fraction (*X*_1_**)**, ideal mole fraction ($X_{1}^{\mathrm{ideal}}),$ interaction parameter (*β*), activity coefficients (***f*_1_, *f*_2_)** of DPH + SDS/CPC mixed (systems at temperature *T* = 298.15 K. Values are the mean ± SD (*n* = 3).

α_1_	CMC (mM)	CMC* (mM)	*X*_1_	$X_{1}^{\mathrm{ideal}}$	−*β*	*f*_1_	*f*_2_	$ln\left(\frac{CMC_{1}}{CMC_{2}}\right)$
SDS+DPH								
0	112 ± 6.10							
0.1	7.877 ± 1.29	45.5	0.534	0.635	7.272	0.206	0.126	−2.75
0.2	6.622 ± 1.21	28.5	0.584	0.796	6.635	0.317	0.104	
0.8	1.239 ± 0.58	8.80	0.647	0.984	12.363	0.214	0.006	
0.9	1.010 ± 0.63	7.89	0.657	0.993	13.961	0.193	0.002	
1	7.150 ± 0.87							
CPC+DPH								
0	112 ± 6.1							
0.1	4.416 ± 1.17	7.65	0.728	0.938	3.988	0.744	0.121	−4.92
0.3	1.890 ± 1.12	2.67	0.811	0.983	4.317	0.857	0.058	
0.5	1.037 ± 1.03	1.62	0.802	0.993	5.938	0.793	0.022	
0.7	0.906 ± 1.05	1.16	0.863	0.997	5.518	0.901	0.016	
0.9	0.703 ± 0.91	0.905	0.872	0.999	7.133	0.890	0.004	
1	0.815 ± 0.23							

**Table 2:** Surface parameters of DPH and SDS/CPC mixed systems at temperature *T* = 298.15 K. Values are the mean ± SD (*n* = 3).

α_1_	10^6^ *Γ*_max_ (mol·m^−2^)	*A*_min_ (nm^2^)	*A*_ideal_ (nm^2^)	*pC*_20_	γ_cmc_ (mN·m^−1^)	π_cmc_ (mN·m^−1^)
SDS+DPH						
0	1.809 ± 0.21	0.917 ± 0.10		0.963 ± 0.08	49.710 ± 0.67	20.290 ± 0.67
0.1	2.213 ± 0.28	0.750 ± 0.09	0.710	2.909 ± 0.14	31.350 ± 0.81	38.650 ± 0.81
0.2	2.257 ± 0.25	0.735 ± 0.13	0.699	3.061 ± 0.09	31.495 ± 0.90	38.505 ± 0.90
0.8	2.277 ± 0.27	0.729 ± 0.11	0.681	3.688 ± 0.11	30.196 ± 0.73	39.804 ± 0.73
0.9	2.328 ± 0.21	0.713 ± 0.06	0.679	3.818 ± 0.07	30.124 ± 0.43	39.876 ± 0.43
1	3.011 ± 0.23	0.551 ± 0.04		2.700 ± 0.05	30.208 ± 0.29	39.792 ± 0.29
CPC+DPH						
0	1.809 ± 0.21	0.917 ± 0.10		0.963 ± 0.08	49.710 ± 0.67	20.290 ± 0.67
0.1	1.219 ± 0.23	1.361 ± 0.09	0.866	3.022 ± 0.09	35.859 ± 0.69	34.141 ± 0.69
0.3	1.243 ± 0.28	1.335 ± 0.15	0.864	3.410 ± 0.13	35.502 ± 0.73	34.498 ± 0.73
0.5	1.541 ± 0.19	1.077 ± 0.12	0.863	3.680 ± 0.11	35.362 ± 0.48	34.638 ± 0.48
0.7	1.266 ± 0.14	1.311 ± 0.07	0.859	3.718 ± 0.15	34.929 ± 0.33	35.071 ± 0.33
0.9	1.109 ± 0.22	1.496 ± 0.09	0.861	3.913 ± 0.08	35.285 ± 0.18	34.715 ± 0.18
1	1.933 ± 0.11	0.858 ± 0.05		3.920 ± 0.11	37.710 ± 0.15	32.290 ± 0.15

**Table 3:** Molar concentrations (*C*^S^), micellar mole fraction at surface (*X*_1_^S^), interaction parameter at surface (*β*^S^), packing parameter (*p*) and CMC/*C*_20_ of DPH and SDS/CPC mixed systems at temperature *T* = 298.15 K. Values are the mean ± SD (*n* = 3).

α_1_	*C*^S^ (mmol·dm^−3^)	*X*_1_^S^	*−β* ^S^	*P*	$\ln\left(\frac{C_{1}}{C_{2}}\right)$
SDS+DPH					
0	109 ± 11			0.23	–3.98
0.1	1.23 ± 6.5	0.565	11.82	0.28	
0.2	0.869 ± 8.3	0.594	11.78	0.29	
0.8	0.205 ± 3.1	0.645	16.52	0.29	
0.9	0.152 ± 1.3	0.650	18.54	0.30	
1	2.04 ± 0.15			0.38	
CPC+DPH					
0	109 ± 11			0.23	–6.81
0.1	0.951 ± 6.8	0.860	4.17	0.15	
0.3	0.341 ± 7.1	0.897	4.79	0.16	
0.5	0.207 ± 3.2	0.907	5.82	0.20	
0.7	0.167 ± 0.23	0.976	4.33	0.16	
0.9	0.122 ± 0.35	0.940	7.49	0.14	
1	0.120 ± 0.17			0.24	

**Table 4:** Thermodynamic parameters of DPH and SDS/CPC mixed at temperature *T* = 298.15 K. Values are the mean ± SD (*n* = 3).

α_1_	−$∆G_{m}^{o}$ (kj·mol^−1^)	−Δ*G*_ads_ (kj·mol^−1^)	*G*_min_ (kj·mol^−1^)	−*G*_EX_
SDS+DPH				
0	15.37 ± 0.15	26.58 ± 0.14	27.46 ± 2.67	
0.1	21.95 ± 0.18	39.41 ± 0.16	14.16 ± 2.35	4.484
0.2	22.38 ± 0.23	39.44 ± 0.19	13.94 ± 1.90	3.994
0.8	26.53 ± 0.27	44.01 ± 0.12	13.25 ± 2.08	6.996
0.9	27.04 ± 0.20	44.17 ± 0.24	12.93 ± 1.67	7.794
1	22.19 ± 0.33	35.41 ± 0.81	10.03 ± 0.90	
CPC+DPH				
0	15.37 ± 0.15	26.58 ± 0.14	27.46 ± 2.67	
0.1	23.38 ± 0.18	51.37 ± 0.80	29.39 ± 2.31	1.957
0.3	25.49 ± 0.15	53.23 ± 0.18	28.54 ± 2.39	1.637
0.5	26.97 ± 0.35	49.45 ± 0.99	22.93 ± 0.99	2.333
0.7	27.31 ± 0.31	55.01 ± 1.01	27.58 ± 1.50	1.616
0.9	27.94 ± 0.17	59.23 ± 0.88	31.80 ± 0.99	1.969
1	27.57 ± 0.57	44.27 ± 1.34	19.49 ± 1.18	

## Data Availability

Not applicable.
